# Roles of c-Met and RON kinases in tumor progression and their potential as therapeutic targets

**DOI:** 10.18632/oncotarget.3420

**Published:** 2015-01-31

**Authors:** Katherine Chang, Anand Karnad, Shujie Zhao, James W. Freeman

**Affiliations:** ^1^ Department of Medicine, Division of Medical Oncology, University of Texas Health Science Center at San Antonio, San Antonio, TX, USA; ^2^ Cancer Therapy and Research Center, Experimental and Developmental Therapeutics Program, San Antonio, TX, USA; ^3^ Research and Development, Audie Murphy Veterans Administration Hospital, San Antonio, TX, USA

**Keywords:** c-Met, RON kinase, Met inhibitors

## Abstract

c-Met and receptor originated from nantes (RON) are structurally related transmembrane phosphotyrosine kinase receptors. c-Met and RON show increased expression or activity in a variety of tumors leading to tumor progression and may play a role in acquired resistance to therapy. Although often co-expressed, the distinct functional roles of c-Met and RON are not fully understood. c-Met and RON form both activated homodimers and heterodimers with themselves and other families of phosphotyrosine kinase receptors. Inhibitors for c-Met and RON including small molecular weigh kinase inhibitors and neutralizing antibodies are in pre-clinical investigation and clinical trials. Several of the tyrosine kinase inhibitors have activity against both c-Met and RON kinases whereas the antibodies generally are target specific. As with many targeted agents used to treat solid tumors, it is likely that c-Met/RON inhibitors will have greater benefit when used in combination with chemotherapy or other targeted agents. A careful analysis of c-Met/RON expression or activity and a better elucidation of how they influence cell signaling will be useful in predicting which tumors respond best to these inhibitors as well as determining which agents can be used with these inhibitors for combined therapy.

## INTRODUCTION

c-Met and RON are structurally related proto-oncogenes belonging to the semaphorin family of transmembrane receptor tyrosine kinases (RTKs) [1]. The semaphorin superfamily are composed of three protein families, the semaphorins, plexins and the c-Met family [2]. c-Met and RON have essential functional roles in embryonic development and organogenesis [3, 4] and are over expressed and/or aberrantly activated in various cancer types suggesting their potential importance as therapeutic targets [5-10]. Evidence points to a role for c-Met and RON signaling in tumor progression by increasing proliferation, inhibiting apoptosis, contributing to angiogenesis, promoting metastasis and in maintenance of cancer stem cells [11-14]. Aberrant expression and activities of c-Met and RON in cancer are attributed to various mechanisms including increased expression of their ligands or receptors and by activating mutations [4, 15]. Over expression of c-Met but seldom RON is linked to gene amplification [16-18]. Mutations in the RON and c-Met promoters are known to enhance transcription and point mutations have been identified that enhance tyrosine kinase activity [19-21]. Pro-tumorigenic activities of RON are also attributed to different isoforms identified in cancer cells. At least six isoform variants of RON are known and these likely originate by alternative pre-mRNA processing, alternative transcription or by truncation [4]. Thus, a variety of mechanisms account for increased expression and/or activity of c-Met and RON in cancer cells. This aberrant expression and activity of c-Met and RON suggest that they are important targets for cancer therapy. Indeed, agents targeting c-Met and RON for cancer therapy are FDA approved or are in various phases of clinical trials and/or pre-clinical testing and these include small molecular weight kinase inhibitors and neutralizing antibodies to the receptors or their ligands [3, 22-28]. Although not comprehensive, this review is intended to provide a summary of the biology of c-Met and RON and the current status of drug development to these targets and the results of pre-clinical and clinical trials of these agents.

### STRUCTURE AND FUNCTION OF C-MET AND RON

#### c-Met and RON Receptors

**Figure 1:**
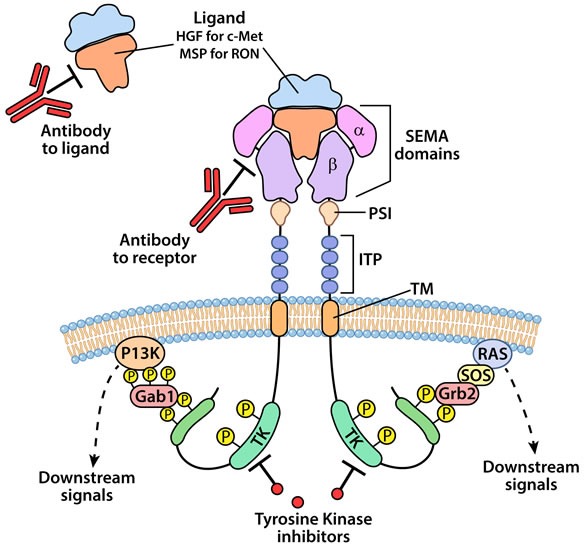
An illustration representing the structural and cell signaling domains and approaches for targeting c-Met and RON kinases for therapy Mature c-Met and RON are structurally similar and are composed of an extracellular α-chain and a β-chain The extracellular domains include the semaphorin (SEMA) that possess ligand binding function, plexin-semaphorin-integrin (PSI) and the immunoglobulin-like plexin transcription (ITP), a transmembrane (TM) and an intracellular tyrosine kinase (TK) domain. Ligand binding, HGF for c-Met and MSP for RON, results in dimerization and phosphorylation in the TK domain leading to conformational changes and autophosphorylation of the C-terminal end of the receptor. The C-terminal phosphorylation of the receptor recruits adaptor proteins generally Gab1 for RON and Grb2 for c-Met which in turn leads to activation of various signaling cascades including PI3K/AKT and Ras/MAPK. Current strategies for targeting c-Met and RON signaling include neutralizing antibodies to the receptors or their ligands and small molecular weight tyrosine kinase inhibitors.

The mature forms of c-Met and RON are approximately 180 kD heterodimeric proteins composed of an extracellular 35 kD α-chain and a 145 kD transmembrane β-chain linked by disulfide bonds. RON shares 25% homology with c-Met in its extracellular domain and 63% homology within the tyrosine kinase domain [29]. c-Met and RON possess remarkably similar functional domains. Both possess an N-terminal SEMA domain that contains the ligand-binding domain and an adjacent cysteine rich domain (CDR), [2, 29]. The extracellular portion of the β-chain possesses a plexins-semaphorin-integrin domain (PSI) and several immunoglobulin-plexin-transcription (IPT) domains [30]. Activation of the intracellular tyrosine kinase domain for c-Met and RON are mediated through phosphorylation of tyrosine residues 1234 and 1235 for c-Met and residues 1238 and 1239 for RON. The activation of the kinase domain is followed by phosphorylation in the carboxy-terminal end at tyrosine residues 1349 and 1356 for c-Met and 1353 and 1360 for RON. These latter phosphorylated residues provide docking sites for adaptor and intracellular kinases that regulate cell signaling cascades. Using site directed mutagenesis, Chaudhuri et al. [31] showed that Tyr-1353 but not Tyr-1360 was necessary for cell signaling for RON. As described in more detail below, Grb-2 appears to be the major adaptor protein that mediates signaling through c-Met; whereas, Gab1 but not Grb2 facilitates signaling by RON. The structural and functional domains of c-Met and RON are illustrated in Figure 1.

#### c-Met and RON ligands

Hepatocyte growth factor (HGF) and macrophage stimulating protein (MSP) are ligands that activate c-Met and RON, respectively [29, 32, 33]. HGF is expressed by multiple tissue types including smooth muscle, fibroblasts, adipose tissue as well as by epithelial derived tumors [34, 35]. HGF was discovered in 1984 as a mitogenic protein for hepatocytes [36] and in 1991 was identified as the ligand for c-Met [37]. HGF is biosynthesized as a pre-pro form of 728 amino acids containing α and β chains and these are subsequently cleaved in several steps to form the active ligand [38]. The α chain of HGF binds to the Sema domain of c-Met with high affinity but activation of c-Met requires the additional binding of the β chain which binds c-Met with low affinity [38].

MSP shares a high level of sequence and structural homology with HGF [39]. MSP is expressed by the liver, lungs, adrenal glands, placenta and kidney and its expression is regulated mainly at the transcriptional level [30]. As with HGF, MSP is secreted as an inactive single chain that is subsequently activated by proteolytic cleavage yielding a dimeric peptide possessing α and β chains. In contrast to HGF, the high affinity RON binding site, for MSP, lies in the β chain. The induction of specific signaling pathways following ligand activation of c-Met or RON is dependent on tissue availability of adapter proteins and signaling intermediates and receptor modulation reflected by homo and heterodimerization.

### CELL SIGNALING BY c-MET AND RON

#### Modulation of phosphotyrosine kinase receptor signaling by interactions of c-Met and RON

RON and c-Met are reported to be co-expressed in many tumor types [40-42] and cross talk between these two receptor pathways is known to occur [43]. Their structural homology suggests that they may interact and indeed recent studies, including our own, indicate that c-Met and RON can form heterodimers and can transphosphorylate one another [44]. A study in four different tumor cell lines showed that oncogenic addiction to c-Met requires co-expression of RON [29]. In this scenario RON was constitutively activated and this constant activation of RON was dependent on transphosphorylation of RON by c-Met [29]. In each of these four cell lines the *c-Met* gene but not the *RON* gene was amplified. Experimentally, c-Met is shown to have stronger kinase activity than RON [45] and thus it is possible that c-Met may be more efficient at activating RON than RON-RON homodimers. The requirement of RON for oncogenic addiction to c-Met implies that c-Met-RON heterodimers promote different signaling cascades because of diverse platforms. However, c-Met and RON possess remarkably similar tyrosine binding sites that serves as docking sites for adaptor or signaling molecules and thus the signaling platforms may be redundant. This appears to not be the case given their differences in strengths as kinases and the recent finding that Grb2 binds directly and is responsible for the biologic activity of c-Met; whereas, RON relies chiefly on Gab1; whereas, Gab2 binding to RON attenuates Gab1 recruitment and represses signaling [31].

As is the case with heterodimers from the EGFR family of receptors, signaling from heterodimers creates signaling diversity. Thus, depending on the relative abundance of each receptor type RON expression may in part modulate c-Met activity and vice versa. In this context, we recently showed that knockdown of RON enhanced the level and duration of HGF mediated activation of MAPK and AKT [44]. The functional relevance of c-Met-RON heterodimers has not been fully investigated. However, two separate studies suggest that genetic knock down of RON leads to up regulation on c-Met signaling [44, 46]. Thus, separately inhibiting either of these receptor kinases may lead to compensation by the other.

Studies also indicate that c-Met and RON may interact with other phosphotyrosine kinases. Lowy and his colleagues recently showed that MSP stimulated RON was unable to activate IGF1-R but that IGF1 or EGF treatment caused phosphorylation of RON [47, 48]. Thus IGF1-R activation of Ron was unidirectional. In contrast, MSP was able to phosphorylate both c-Met and EGFR in a RON dependent manner and activated RON was co-immunoprecipitated with each of these receptors [47, 48]. Similarly c-Met is known to activate IGF1-R [5]. However, activation of c-Met or RON by IGF or EGF is relatively weak and the significance of this *in vivo* is yet to be firmly established. A separate study showed that activated EGFR is able to phosphorylate c-Met indirectly through Src [49]. Regardless of the mechanisms, c-Met and RON are likely to modulate signaling by direct or indirect interaction with other phosphotyrosine kinase receptors.

#### Pathways activated and biologic consequence of c-Met and Ron activation

**Figure 2:**
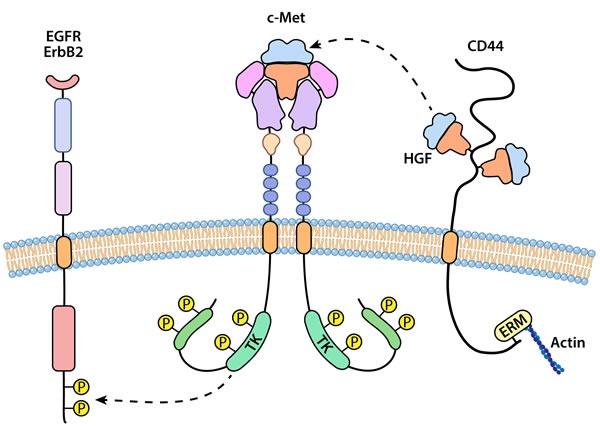
An illustration representing interaction of c-Met or RON with other cell surface receptors Homodimerization of c-Met or RON appears preferable although c-Met and RON can form heterodimers leading to transphosphorylation. c-Met and RON may interact with and transphosphorylate other receptor tyrosine kinases including members of the EGFR family. A separate type of interaction for c-Met is with CD44, a non-kinase transmembrane receptor. Isoforms of CD44 bind and apparently sequester HGF at the membrane, acting a co-receptor for presentation of ligand to c-Met.

The recruitment and binding of substrates/adaptor proteins to the phosphorylated carboxy-terminal docking sites of activated c-Met and RON provides the platform to activate signaling cascades. As described above, the docking sites are Tyr-1349 and Tyr-1356 for c-Met and Tyr-1353 and Tyr-1360 for RON. Potential signaling cascades are illustrated in Figure 2 and most appear dependent on PI3K and MAPK activation as central switches. Major signaling molecules activated through c-Met and RON signaling include MAPK, PI3K/AKT, c-Src, STAT3, NF-κB, FAK and β-catenin and most of these may be dependent on PI3K and MAPK. The mediators of c-Src and STAT3 by c-Met and RON are not fully determined although JAK inhibitors blocked STAT3 activation by HGF stimulation in some cell lines suggesting that JAK could interact directly or indirectly with c-Met. These activated signaling molecules in turn govern the cellular responses to activated c-Met or RON.

Numerous cellular responses are attributed to c-Met and RON signaling and these induce but are not limited to cytoskeletal changes, EMT, migration and invasion, stemness, resistance to apoptosis, angiogenesis and proliferation. It is likely that activation of down stream molecular targets and subsequent biologic responses are cell context dependent and require critical levels of adaptor and singling molecules and are highly dependent on cross talk with other signaling molecules. c-Met can directly interact with E-cadherin, disrupting adherens junctions and leading to nuclear accumulation of β-catenin which potentially drives EMT in epithelial derived tumors [50, 51]. Similarly, RON signaling is known to activate HIF-1α down stream of mTOR [52]. The stem cell marker and hyaluronan receptor CD44 modulates c-Met signaling by several mechanism, first by acting as a co-receptors for HGF [53, 54] and second by interacting directly with E-cadherin and forming a complex with ERM proteins and the actin cytoskeleton [54]. c-Met and RON signaling stimulates angiogenesis primarily by inducing VEGF likely through up regulation of HIF-1α. RON signaling was known to activate HIF-1α down stream of mTOR [52, 55]. Both RON and c-Met signaling are reported to activate STAT3 [46, 55] and it is possible that this may JAK dependent although other tyrosine kinases including Src and c-Abl may play roles depending on the cell type. RON and c-Met signaling may mediate resistance to apoptosis through multiple pathways. A study by Logan-Collins et al [46] showed that overexpression of Ron lead to up regulation of anti-apoptotic molecules including bcl2. Similarly, inhibition of c-Met signaling increases mitochondrial release of cytochrome C and increased the Bax/bcl2 ratio [56]. Thus, c-Met and Ron regulate a number of pro-tumorigenic pathways supporting their potential value as therapeutic target.

#### Role of c-Met and RON in maintenance of cancer stem cells

**Figure 3:**
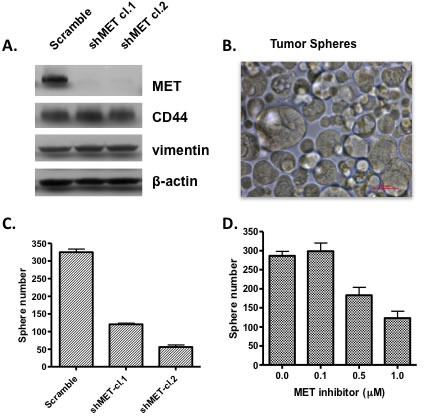
Inhibiting c-Met expression or activity prevents growth of tumor spheres Cells from CFPac-1 pancreatic cancer cell line were separated by flow cytometry on the basis of high CD44 expression. The high CD44 expressing cell population show high expression of c-Met and where able to grow after repeated passages as tumor spheres. (A) c-Met was knocked down using a shRNA approach in high CD44 expressing pancreatic cancer cells; (B) photo showing that CD44 high expressing cells were able to grow as tumor spheres in stem cell medium; (C) Knockdown of c-Met by shRNA inhibited the ability of high CD44 expressing pancreatic cancer cells to grow as tumor spheres and (D) treatment of high CD44 expressing cancer cells with a c-Met tyrosine kinase inhibitor prevented their growth as tumor spheres.

There is increasing evidence that c-Met is involved in expansion and maintenance of cancer stem cells. However, evidence of RON signaling in stemness is not clear. The function of c-Met in maintenance of cancer stem cell phenotype is consistent with its role in embryogenesis and tissue development. The suggestion that c-Met was involved in stem cells has been around for a number of years including a study by Kmiecik et al [57] who showed that HGF and c-Met expression were required for colony formation of hematopoietic progenitor cells. The requisite for c-Met for the cancer stem cell phenotype is clearly established for glioblastomas (GBM), [58, 59]. More recently, Boccaccio and colleagues [6] showed that c-Met signaling was a crucial regulator of the genetic program related to EMT, invasive growth and maintenance of a GBM stem cell phenotype. A study in head and neck squamous cell carcinoma (HNSCC) show that c-Met positive cells display cancer stem cell properties and are responsible for resistance to cisplatin [6]. Similarly, prostate cancer stem-like cells expressed c-Met and HGF/c-Met signaling was required in these cells for self renewal [60]. High expression of c-Met was also showed to be found in pancreatic cancer stem cells [15, 61] and as shown here, knockdown of c-Met or treatment with a c-Met inhibitor blocked the ability to form tumor spheres in a population of pancreatic cancer cells with stem cell like properties (Figure 3). The role of RON in maintenance of a cancer stem cell phenotype is not fully studied. Sustained expression of RON was reported for a pancreatic stem cell like population of cells isolated from the L3.6pl cell line [61]. It is possible that inhibiting both RON and c-Met will more fully eliminate the cancer stem cell population. This further suggests that inhibitors that block kinases activities of both c-Met and RON may be preferable over specific agents; especially where both kinases are co-expressed.

### c-MET AND RON AS MOLECULAR TARGETS

#### Pre-clinical and clinical studies

c-Met and RON are over expressed in many cancer types leading to aberrant signaling that contributes to cancer development and progression [30, 62]. MET expression is associated with worse clinical prognosis and aggression in different tumor types [33, 63-66]. These findings lead to strategies for targeting c-Met and RON for cancer therapy. Preclinical studies as reviewed by Wang and colleagues [27] indicate that c-Met and RON are clinically relevant therapeutic targets in multiple types of cancers. For example, MET abnormalities have been reported in a small number of patients with gastroesophageal adenocarcinoma, although prognostic and predictive implications of this remains to be investigated [67]. Most studies focus on inhibiting either c-Met or RON separately, although, as discussed below, many of the current small molecular weight tyrosine kinase inhibitors (TKIs) developed against c-Met have varying levels of activity against RON. Others and we [8, 14, 46, 68] have reported that RON is over expressed in pancreatic cancers. A recent study showed that RON is increasingly expressed during progression of pancreatic cancer (68) and that RON shows sustained expression in pancreatic cancer stem cells [69] suggesting its potential value as a therapeutic target for this disease. Moreover, inhibiting RON expression suppressed growth of pancreatic cancer orthotopic implants [44, 46]and increased sensitivity to gemcitabine [46]. Genetic knockdown of RON by itself delayed but did not prevent tumor progression and resulting tumors showed increased activation of c-Met which is generally co-expressed with RON in pancreatic cancer cell lines [44]. Thus, it is likely that therapeutic strategies designed to specifically target c-Met or RON could be problematic due to compensatory mechanisms caused by increased activity by the other receptor.

The major successes of biologic targeted therapies along with preclinical findings showing that c-Met and RON are aberrantly expressed in cancer cell lines and tissues led to efforts to develop agents that inhibit their function or activities. As with other tyrosine kinase receptors, various approaches are being investigated for inhibiting c-Met and RON. These approaches include natural inhibitors of ligand binding, ribozymes and siRNAs, decoy receptors that capture ligand, neutralizing antibodies to ligands or antibodies to the receptors that block signaling and small molecular weight tyrosine kinase inhibitors (TKIs). These approaches and preclinical testing are detailed in numerous reviews including [10, 27, 30, 70, 71]. The strategies receiving the most attention and that have moved onto clinical trials are humanized monoclonal antibodies to the ligands or receptors and TKIs. A partial list of agents currently approved or in various phases of clinical trials is provided in Table 1. A brief but not comprehensive description of a few of these is provided below. It should be noted that strategies to target c-Met are further along than those for RON. However, because of structural homologies in their kinase domains, many of the TKIs generated for c-Met show activity against RON as mentioned above. Moreover, several of these TKIs are considered multi-kinase inhibitors and have activities against tyrosine kinases belonging to other families.

**Table 1:** List of agents that target c-Met and/or RON

Agent -	Type	Target**	Status	Tumor Type
Cabozanilnib *(XL 184)*	(TKI)	c-Met VEGFR2/RET	FDA approved	PMMTC
Tivantinib *(4GI97)*	(TKI)	c-Met	Phase Il/Ill	HCC, PSC
HNSCC				
INC 280	(TKI)	c-Met	Phase I/lI	GB, NSCLC, RCC
MSC21561 19J	(TKI)	c-Met	Phase II	NSCLC, HCC
LGx8I8	(TKI)	c-Met	Phase II	ML
AMG337	(TKI)	c-Met	Phase I	solid tumors
LY280 1653	(TKI)	c-Met/RON	Phase I	NSCLC
Foretinib *(GSK1363089)*	(TKI)	c-Met/VEGFR2/RON	Phase II	GC, RCC
Golvatinib *(E7050)*	(TKI)	c-Met/VEGFR	Phase I/Il	Solid tumors
MGCD265	(TKI)	c-MET/VEGFR	Phase I/II	Solid tumors
Rilotumumab *(AMG102)*	(MAb)	HGF	Phase II	0V
Narnatumab *(IMC-Ron8)*	(MAb)	RON	Phase I	solid tumors

* partial list,

** TKIs against c-Met may show varying level of activity against RON

TKI, tyrosine kinase inhibitor; MAb, monoclonal antibody; PMMTC, progressive metastatic medullary thyroid cancer; HCC, hepatocellular carcinoma; PSC, prostate cancer; HNSCC, head and neck squamous cell carcinoma; GB, glioblastoma; RCC, renal cell carcinoma; NSCLC, non small cell lung cancer; ML, melanoma; OV, ovarian; HGF, hepatocyte growth factor.

Tivantinib (originally called ARQ 197) a c-Met TKI has gone through phase II trials as a single agent or in combination with other targeted agents or chemotherapy [72]. Based on encouraging phase II studies, phase III trials are also underway. Phase II studies using tivantinib as a single agent second line therapy in advanced hepatocellular carcinoma (HCC) showed the most significant benefit was obtained for patients expressing high levels of c-Met [73]. High levels of c-Met was defined as greater than 50 % of tumors showing 2 to 3 plus level of staining by immunohistochemistry (IHC) where 1 plus represents weak staining. In the c-Met high group, there was an increase in overall survival (OS) from 3.8 months in placebo to 7.2 months in the tivantinib treated group. The overexpression of c-Met in HCC has been reported to be near 30% [74]. There has been a great deal of interest in targeting c-Met in non-squamous NSCLC since 76% are reported to over express c-Met[75] and that c-Met may cause resistance to EGFR inhibitors.[76, 77]. A phase II study in NSCLC showed that a combination of tivantinib and erlotinib increased progression free survival (PFS) compared to erlotinib alone [78]. A follow-up phase III MARQUEE trial, did not reach primary endpoint of prolonging overall survival at interim analysis with addition of tivantinib to erlotinib, and so the study was halted. However, there was improvement in progression-free survival (3.6 vs. 1.9 months, p<0.0001) and overall response rate (10.3% vs. 6.5%, p<0.05), with similar tolerance and safety profiles. Molecular subgroup analysis, including MET expression is ongoing [79]. A phase II clinical trial for Foretinib (also called XL880) in papillary renal cell carcinoma (pRCC) showed an overall response rate (ORR) of only 13.5% but an ORR of 50% in patients with a c-Met germ line mutation; however, c-Met amplification was found in only 3% for this tumor type [80]. Crizotinib has been approved for the treatment of metastatic ALK-positive non-small cell lung cancer, but it is also a potent MET inhibitor and is undergoing phase I studies in patients with c-Met positive tumors [76, 81].

#### Role of c-Met and RON in promoting resistance to anti-cancer agents

A number of recent studies, as described below, have linked aberrant activity of c-Met or RON with resistance of tumor cells to cancer therapies. These studies imply that up regulated signaling through c-Met and RON may be induced in response to chemotherapy or biologically targeted therapy. For example, amplification of MET has been associated with EGFR TKI resistance. Patients who harbor EGFR mutations get treated with erlotinib or gefinitib, and will invariably develop TKI resistance, and ~20% of which can be associated with MET amplification [82]. It has been suggested that targeting MET may be useful in patients with acquired resistance to TKI therapy [16]. In most instances studies examining the roles of either c-Met or RON have not looked at the influence of c-Met and RON together. We found that shRNA knockdown of RON in pancreatic cancer cell lines leads to up regulation in expression and activation of c-Met, suggesting the need to co-target or to use an agent that inhibits both of these kinases [44]. There is an accumulation of data supporting a role of c-Met as a mediator in resistance to cancer therapies as reviewed by Maroun and Rowlands [70]. RON, although not as thoroughly studied, is also implicated in resistance to anti-cancer agents [11, 46, 83, 84]. It is not possible to review each of these studies here. An interesting and representative example of how c-Met activation mediates resistance to anti-VEGF therapies is described by Matsumara and colleagues [85]. The role of c-Met in mediating resistance to VEGF-pathways is of particular importance because of prominence of targeting VEGF signaling for anti-angiogenic therapy. To our knowledge, whether RON plays a similar role in promoting resistance to anti-angiogenic therapy has not been investigated.

Targeting VEGF or VEGFR represents the first major success in the clinic for inhibiting angiogenesis. Bevacizumab, a humanized anti-VEGF antibody, is approved for metastatic colorectal and renal cell carcinomas and for glioblastomas and non-small cell lung cancers. Tyrosine kinase inhibitors (TKIs) that target VEGFR with various levels of specificity including sunitinib, sorafenib, pazopanib, tivozanib, axitinib, cabozantinib and dovitinib are approved or in clinical trials as reviewed [86, 87]. However, the clinical benefit of VEGF/VEGFR based anti-angiogenic therapies is limited because of development of resistance. A number of preclinical studies including [88, 89] show that despite anti-tumor activity continued treatment with inhibitors of VEGF pathways increased invasion and malignant progression. Subsequent to these findings, studies by Sennino and colleagues [90] implicate the up regulation of c-Met in resistance to VEGF pathway targeted therapies. In this study Sennino and his colleagues used two separate models to show that continued treatment with anti-VEGF pathway therapies leads to regression of the primary tumor and increased survival times. However, this treatment also induced a phenotypic change in a subpopulation of tumor cells that resulted in increased invasion and metastasis. The mechanism for this phenotypic switch and increased invasion appears to be mediated by HIF1α induced c-Met expression. In this context HIF1α was induced by hypoxia resulting from continued treatment with VEGF pathway inhibitors. Moreover, this study went on to show that selective inhibitors of c-Met were able to reverse this phenotypic switch and prevented the invasive phenotype induced by treatment with VEGF pathway inhibitors [90]. This study was restricted to an orthotopic model of pancreatic adenocarcinoma and a pancreatic neuroendocrine tumor model. It remains to be determined how general this mechanism may be in relation to other tumor types. However these findings support the use of combined targeting of the VEGF and c-Met pathways in tumors where inhibitors of VEGF pathway caused up regulation of c-Met.

## CONCLUSIONS

C-met and RON are structurally related tyrosine kinase receptors that contribute to tumor progression and promote resistance to chemotherapy. Further understanding of the pathogenesis and signaling pathways of these receptors may provide valuable insight into their role in cancer development and progression of disease. C-Met and RON are activated by separate ligands and following ligand activation they can homo- or heterodimerize with each other. Moreover, they may interact with and modulate signaling of other receptor tyrosine kinases. Expression of these receptors are associated with worse prognosis in various tumor types, and increasing expression of them have been seen in the progression of aggressive tumors. Moreover, c-Met and possibly RON are associated with maintenance of cancer stem cells. As a result, there continues to be advances in the study of these receptors and their signaling pathways with respect to cancer drug development. Numerous clinical trials are in progress using tyrosine kinase inhibitors directed at these receptors as potential molecular targets, with promising results. More studies remain to be seen if these drugs are effective and safe in improving patient survival and tumor response rates, as well as preventing development of tumor resistance to chemotherapy.

## References

[1] Wang MH, Zhang R, Zhou YQ, Yao HP (2013). Pathogenesis of RON receptor tyrosine kinase in cancer cells: activation mechanism, functional crosstalk, and signaling addiction. Journal of biomedical research.

[2] Gherardi E, Love CA, Esnouf RM, Jones EY (2004). The sema domain. Current opinion in structural biology.

[3] Liu X, Newton RC, Scherle PA (2010). Developing c-MET pathway inhibitors for cancer therapy: progress and challenges. Trends in molecular medicine.

[4] Lu Y, Yao HP, Wang MH (2007). Multiple variants of the RON receptor tyrosine kinase: biochemical properties, tumorigenic activities, and potential drug targets. Cancer letters.

[5] Benvenuti S, Comoglio PM (2007). The MET receptor tyrosine kinase in invasion and metastasis. Journal of cellular physiology.

[6] Boccaccio C, Comoglio PM (2013). The MET oncogene in glioblastoma stem cells: implications as a diagnostic marker and a therapeutic target. Cancer research.

[7] Camp ER, Liu W, Fan F, Yang A, Somcio R, Ellis LM (2005). RON, a tyrosine kinase receptor involved in tumor progression and metastasis. Annals of surgical oncology.

[8] Camp ER, Yang A, Gray MJ, Fan F, Hamilton SR, Evans DB (2007). Tyrosine kinase receptor RON in human pancreatic cancer: expression, function, and validation as a target. Cancer.

[9] Graveel CR, Tolbert D, Vande Woude GF (2013). MET: a critical player in tumorigenesis and therapeutic target. Cold Spring Harbor perspectives in biology.

[10] Kang CM, Babicky ML, Lowy AM (2014). The RON receptor tyrosine kinase in pancreatic cancer pathogenesis and its potential implications for future targeted therapies. Pancreas.

[11] McClaine RJ, Marshall AM, Wagh PK, Waltz SE (2010). Ron receptor tyrosine kinase activation confers resistance to tamoxifen in breast cancer cell lines. Neoplasia.

[12] Previdi S, Abbadessa G, Dalo F, France DS, Broggini M (2011). Breast Cancer-Derived Bone Metastasis can be Effectively Reduced through Specific c-MET Inhibitor Tivantinib (ARQ 197) and shRNA c-MET knockdown. Molecular cancer therapeutics.

[13] Takeuchi H, Bilchik A, Saha S, Turner R, Wiese D, Tanaka M (2003). c-MET expression level in primary colon cancer: a predictor of tumor invasion and lymph node metastases. Clinical cancer research : an official journal of the American Association for Cancer Research.

[14] Zhao S, Ammanamanchi S, Brattain M, Cao L, Thangasamy A, Wang J (2008). Smad4-dependent TGF-beta signaling suppresses RON receptor tyrosine kinase-dependent motility and invasion of pancreatic cancer cells. The Journal of biological chemistry.

[15] Li C, Wu JJ, Hynes M, Dosch J, Sarkar B, Welling TH (2011). c-Met is a marker of pancreatic cancer stem cells and therapeutic target. Gastroenterology.

[16] Engelman JA, Zejnullahu K, Mitsudomi T, Song Y, Hyland C, Park JO (2007). MET amplification leads to gefitinib resistance in lung cancer by activating ERBB3 signaling. Science.

[17] Han CB, Ma JT, Li F, Zhao JZ, Jing W, Zhou Y (2011). EGFR and KRAS mutations and altered c-Met gene copy numbers in primary non-small cell lung cancer and associated stage N2 lymph node-metastasis. Cancer letters.

[18] Zeng ZS, Weiser MR, Kuntz E, Chen CT, Khan SA, Forslund A (2008). c-Met gene amplification is associated with advanced stage colorectal cancer and liver metastases. Cancer letters.

[19] Peace BE, Hughes MJ, Degen SJ, Waltz SE (2001). Point mutations and overexpression of Ron induce transformation, tumor formation, and metastasis. Oncogene.

[20] Ma PC, Tretiakova MS, MacKinnon AC, Ramnath N, Johnson C, Dietrich S (2008). Expression and mutational analysis of MET in human solid cancers. Genes, chromosomes & cancer.

[21] Santoro MM, Penengo L, Minetto M, Orecchia S, Cilli M, Gaudino G (1998). Point mutations in the tyrosine kinase domain release the oncogenic and metastatic potential of the Ron receptor. Oncogene.

[22] Christensen JG, Burrows J, Salgia R (2005). c-Met as a target for human cancer and characterization of inhibitors for therapeutic intervention. Cancer letters.

[23] Dussault I, Bellon SF (2009). From concept to reality: the long road to c-Met and RON receptor tyrosine kinase inhibitors for the treatment of cancer. Anti-cancer agents in medicinal chemistry.

[24] Eder JP, Vande Woude GF, Boerner SA, LoRusso PM (2009). Novel therapeutic inhibitors of the c-Met signaling pathway in cancer. Clinical cancer research : an official journal of the American Association for Cancer Research.

[25] Katz JD, Jewell JP, Guerin DJ, Lim J, Dinsmore CJ, Deshmukh SV (2011). Discovery of a 5H-benzo[4,5]cyclohepta[1,2-b]pyridin-5-one (MK-2461) inhibitor of c-Met kinase for the treatment of cancer. Journal of medicinal chemistry.

[26] Li Z, Yao H, Guin S, Padhye SS, Zhou YQ, Wang MH (2010). Monoclonal antibody (mAb)-induced down-regulation of RON receptor tyrosine kinase diminishes tumorigenic activities of colon cancer cells. International journal of oncology.

[27] Wang MH, Padhye SS, Guin S, Ma Q, Zhou YQ (2010). Potential therapeutics specific to c-MET/RON receptor tyrosine kinases for molecular targeting in cancer therapy. Acta pharmacologica Sinica.

[28] Yap TA, Sandhu SK, Alam SM, de Bono JS (2011). HGF/c-MET Targeted Therapeutics: Novel Strategies for Cancer Medicine. Current drug targets.

[29] Benvenuti S, Lazzari L, Arnesano A, Li Chiavi G, Gentile A, Comoglio PM (2011). Ron kinase transphosphorylation sustains MET oncogene addiction. Cancer research.

[30] Yao HP, Zhou YQ, Zhang R, Wang MH (2013). MSP-RON signalling in cancer: pathogenesis and therapeutic potential. Nature reviews Cancer.

[31] Chaudhuri A, Xie MH, Yang B, Mahapatra K, Liu J, Marsters S (2011). Distinct involvement of the Gab1 and Grb2 adaptor proteins in signal transduction by the related receptor tyrosine kinases RON and MET. The Journal of biological chemistry.

[32] Gaudino G, Follenzi A, Naldini L, Collesi C, Santoro M, Gallo KA (1994). RON is a heterodimeric tyrosine kinase receptor activated by the HGF homologue MSP. The EMBO journal.

[33] Mariani M, McHugh M, Petrillo M, Sieber S, He S, Andreoli M (2014). HGF/c-Met axis drives cancer aggressiveness in the neo-adjuvant setting of ovarian cancer. Oncotarget.

[34] Maulik G, Shrikhande A, Kijima T, Ma PC, Morrison PT, Salgia R (2002). Role of the hepatocyte growth factor receptor, c-Met, in oncogenesis and potential for therapeutic inhibition. Cytokine & growth factor reviews.

[35] Chen XP, Ren XP, Lan JY, Chen YG, Shen ZJ (2014). Analysis of HGF, MACC1, C-met and apoptosis-related genes in cervical carcinoma mice. Molecular biology reports.

[36] Nakamura T, Nawa K, Ichihara A (1984). Partial purification and characterization of hepatocyte growth factor from serum of hepatectomized rats. Biochemical and biophysical research communications.

[37] Naldini L, Vigna E, Narsimhan RP, Gaudino G, Zarnegar R, Michalopoulos GK (1991). Hepatocyte growth factor (HGF) stimulates the tyrosine kinase activity of the receptor encoded by the proto-oncogene c-MET. Oncogene.

[38] Nakamura T, Mizuno S (2010). The discovery of hepatocyte growth factor (HGF) and its significance for cell biology, life sciences and clinical medicine. Proceedings of the Japan Academy Series B, Physical and biological sciences.

[39] Donate LE, Gherardi E, Srinivasan N, Sowdhamini R, Aparicio S, Blundell TL (1994). Molecular evolution and domain structure of plasminogen-related growth factors (HGF/SF and HGF1/MSP). Protein science : a publication of the Protein Society.

[40] Comperat E, Roupret M, Chartier-Kastler E, Bitker MO, Richard F, Camparo P (2008). Prognostic value of MET, RON and histoprognostic factors for urothelial carcinoma in the upper urinary tract. The Journal of urology.

[41] Cheng HL, Liu HS, Lin YJ, Chen HH, Hsu PY, Chang TY (2005). Co-expression of RON and MET is a prognostic indicator for patients with transitional-cell carcinoma of the bladder. British journal of cancer.

[42] Maggiora P, Lorenzato A, Fracchioli S, Costa B, Castagnaro M, Arisio R (2003). The RON and MET oncogenes are co-expressed in human ovarian carcinomas and cooperate in activating invasiveness. Experimental cell research.

[43] Follenzi A, Bakovic S, Gual P, Stella MC, Longati P, Comoglio PM (2000). Cross-talk between the proto-oncogenes Met and Ron. Oncogene.

[44] Zhao S, Cao L, Freeman JW (2013). Knockdown of RON receptor kinase delays but does not prevent tumor progression while enhancing HGF/MET signaling in pancreatic cancer cell lines. Oncogenesis.

[45] Santoro MM, Collesi C, Grisendi S, Gaudino G, Comoglio PM (1996). Constitutive activation of the RON gene promotes invasive growth but not transformation. Molecular and cellular biology.

[46] Logan-Collins J, Thomas RM, Yu P, Jaquish D, Mose E, French R (2010). Silencing of RON receptor signaling promotes apoptosis and gemcitabine sensitivity in pancreatic cancers. Cancer research.

[47] Jaquish DV, Yu PT, Shields DJ, French RP, Maruyama KP, Niessen S (2011). IGF1-R signals through the RON receptor to mediate pancreatic cancer cell migration. Carcinogenesis.

[48] Keller J, Nimnual AS, Shroyer KR, Joy C, Ischenko I, Chandler CS (2013). Ron tyrosine kinase receptor synergises with EGFR to confer adverse features in head and neck squamous cell carcinoma. British journal of cancer.

[49] Dulak AM, Gubish CT, Stabile LP, Henry C, Siegfried JM (2011). HGF-independent potentiation of EGFR action by c-Met. Oncogene.

[50] Graveel CR, DeGroot JD, Su Y, Koeman J, Dykema K, Leung S (2009). Met induces diverse mammary carcinomas in mice and is associated with human basal breast cancer. Proceedings of the National Academy of Sciences of the United States of America.

[51] Al Moustafa AE (2013). Epithelial-mesenchymal transition and its regulators are major targets of triple-negative breast cancer. Cell adhesion & migration.

[52] Thangasamy A, Rogge J, Ammanamanchi S (2008). Regulation of RON tyrosine kinase-mediated invasion of breast cancer cells. The Journal of biological chemistry.

[53] Dortet L, Veiga E, Bonazzi M, Cossart P (2010). CD44-independent activation of the Met signaling pathway by HGF and InlB. Microbes and infection /Institut Pasteur.

[54] Orian-Rousseau V, Chen L, Sleeman JP, Herrlich P, Ponta H (2002). CD44 is required for two consecutive steps in HGF/c-Met signaling. Genes & development.

[55] Ni S, Zhao C, Feng GS, Paulson RF, Correll PH (2007). A novel Stat3 binding motif in Gab2 mediates transformation of primary hematopoietic cells by the Stk/Ron receptor tyrosine kinase in response to Friend virus infection. Molecular and cellular biology.

[56] Liu Y, Liu JH, Chai K, Tashiro S, Onodera S, Ikejima T (2013). Inhibition of c-Met promoted apoptosis, autophagy and loss of the mitochondrial transmembrane potential in oridonin-induced A549 lung cancer cells. The Journal of pharmacy and pharmacology.

[57] Kmiecik TE, Keller JR, Rosen E, Vande Woude GF (1992). Hepatocyte growth factor is a synergistic factor for the growth of hematopoietic progenitor cells. Blood.

[58] Joo KM, Jin J, Kim E, Ho Kim K, Kim Y, Gu Kang B (2012). MET signaling regulates glioblastoma stem cells. Cancer research.

[59] Li Y, Li A, Glas M, Lal B, Ying M, Sang Y (2011). c-Met signaling induces a reprogramming network and supports the glioblastoma stem-like phenotype. Proceedings of the National Academy of Sciences of the United States of America.

[60] Nishida S, Hirohashi Y, Torigoe T, Inoue R, Kitamura H, Tanaka T (2013). Prostate cancer stem-like cells/cancer-initiating cells have an autocrine system of hepatocyte growth factor. Cancer science.

[61] Herreros-Villanueva M, Zubia-Olascoaga A, Bujanda L (2012). c-Met in pancreatic cancer stem cells: therapeutic implications. World journal of gastroenterology : WJG.

[62] Ma PC, Tretiakova MS, Nallasura V, Jagadeeswaran R, Husain AN, Salgia R (2007). Downstream signalling and specific inhibition of c-MET/HGF pathway in small cell lung cancer: implications for tumour invasion. British journal of cancer.

[63] Miyata Y, Sagara Y, Kanda S, Hayashi T, Kanetake H (2009). Phosphorylated hepatocyte growth factor receptor/c-Met is associated with tumor growth and prognosis in patients with bladder cancer: correlation with matrix metalloproteinase-2 and -7 and E-cadherin. Human pathology.

[64] Oda Y, Sakamoto A, Saito T, Kinukawa N, Iwamoto Y, Tsuneyoshi M (2000). Expression of hepatocyte growth factor (HGF)/scatter factor and its receptor c-MET correlates with poor prognosis in synovial sarcoma. Human pathology.

[65] Baschnagel AM, Williams L, Hanna A, Chen PY, Krauss DJ, Pruetz BL (2014). c-Met expression is a marker of poor prognosis in patients with locally advanced head and neck squamous cell carcinoma treated with chemoradiation. International journal of radiation oncology, biology, physics.

[66] Xie Q, Su Y, Dykema K, Johnson J, Koeman J, De Giorgi V (2013). Overexpression of HGF Promotes HBV-Induced Hepatocellular Carcinoma Progression and Is an Effective Indicator for Met-Targeting Therapy. Genes & cancer.

[67] Jardim DL, de Melo Gagliato D, Falchook GS, Janku F, Zinner R, Wheler JJ (2014). MET aberrations and c-MET inhibitors in patients with gastric and esophageal cancers in a phase I unit. Oncotarget.

[68] Thomas RM, Jaquish DV, French RP, Lowy AM (2010). The RON tyrosine kinase receptor regulates vascular endothelial growth factor production in pancreatic cancer cells. Pancreas.

[69] Padhye SS, Guin S, Yao HP, Zhou YQ, Zhang R, Wang MH (2011). Sustained Expression of the RON Receptor Tyrosine Kinase by Pancreatic Cancer Stem Cells as a Potential Targeting Moiety for Antibody-Directed Chemotherapeutics. Molecular pharmaceutics.

[70] Maroun CR, Rowlands T (2014). The Met receptor tyrosine kinase: A key player in oncogenesis and drug resistance. Pharmacology & therapeutics.

[71] Medova M, Aebersold DM, Blank-Liss W, Streit B, Medo M, Aebi S (2010). MET Inhibition Results in DNA Breaks and Synergistically Sensitizes Tumor Cells to DNA-Damaging Agents Potentially by Breaching a Damage-Induced Checkpoint Arrest. Genes & cancer.

[72] Rimassa L, Personeni N, Simonelli M, Santoro A (2013). Tivantinib: a new promising mesenchymal-epithelial transition factor inhibitor in the treatment of hepatocellular carcinoma. Future Oncol.

[73] Santoro A, Rimassa L, Borbath I, Daniele B, Salvagni S, Van Laethem JL (2013). Tivantinib for second-line treatment of advanced hepatocellular carcinoma: a randomised, placebo-controlled phase 2 study. The lancet oncology.

[74] Lee SJ, Lee J, Sohn I, Mao M, Kai W, Park CK (2013). A survey of c-MET expression and amplification in 287 patients with hepatocellular carcinoma. Anticancer research.

[75] Cappuzzo F, Marchetti A, Skokan M, Rossi E, Gajapathy S, Felicioni L (2009). Increased MET gene copy number negatively affects survival of surgically resected non-small-cell lung cancer patients. Journal of clinical oncology : official journal of the American Society of Clinical Oncology.

[76] Nanjo S, Yamada T, Nishihara H, Takeuchi S, Sano T, Nakagawa T (2013). Ability of the Met kinase inhibitor crizotinib and new generation EGFR inhibitors to overcome resistance to EGFR inhibitors. PloS one.

[77] Benedettini E, Sholl LM, Peyton M, Reilly J, Ware C, Davis L (2010). Met activation in non-small cell lung cancer is associated with de novo resistance to EGFR inhibitors and the development of brain metastasis. The American journal of pathology.

[78] Sequist LV, von Pawel J, Garmey EG, Akerley WL, Brugger W, Ferrari D (2011). Randomized phase II study of erlotinib plus tivantinib versus erlotinib plus placebo in previously treated non-small-cell lung cancer. Journal of clinical oncology : official journal of the American Society of Clinical Oncology.

[79] Scagliotti GV, Novello S, Schiller JH, Hirsh V, Sequist LV, Soria JC (2012). Rationale and design of MARQUEE: a phase III, randomized, double-blind study of tivantinib plus erlotinib versus placebo plus erlotinib in previously treated patients with locally advanced or metastatic, nonsquamous, non-small-cell lung cancer. Clinical lung cancer.

[80] Choueiri TK, Vaishampayan U, Rosenberg JE, Logan TF, Harzstark AL, Bukowski RM (2013). Phase II and biomarker study of the dual MET/VEGFR2 inhibitor foretinib in patients with papillary renal cell carcinoma. Journal of clinical oncology : official journal of the American Society of Clinical Oncology.

[81] Yan HH, Jung KH, Son MK, Fang Z, Kim SJ, Ryu YL (2014). Crizotinib exhibits antitumor activity by targeting ALK signaling not c-MET in pancreatic cancer. Oncotarget.

[82] Bean J, Brennan C, Shih JY, Riely G, Viale A, Wang L (2007). MET amplification occurs with or without T790M mutations in EGFR mutant lung tumors with acquired resistance to gefitinib or erlotinib. Proceedings of the National Academy of Sciences of the United States of America.

[83] Zou Y, Howell GM, Humphrey LE, Wang J, Brattain MG (2013). Ron knockdown and Ron monoclonal antibody IMC-RON8 sensitize pancreatic cancer to histone deacetylase inhibitors (HDACi). PloS one.

[84] Wang Q, Quan H, Zhao J, Xie C, Wang L, Lou L (2013). RON confers lapatinib resistance in HER2-positive breast cancer cells. Cancer letters.

[85] Matsumura A, Kubota T, Taiyoh H, Fujiwara H, Okamoto K, Ichikawa D (2013). HGF regulates VEGF expression via the c-Met receptor downstream pathways, PI3K/Akt, MAPK and STAT3, in CT26 murine cells. International journal of oncology.

[86] Grunwald V, Merseburger AS (2012). Axitinib for the treatment of patients with advanced metastatic renal cell carcinoma (mRCC) after failure of prior systemic treatment. OncoTargets and therapy.

[87] Funakoshi T, Latif A, Galsky MD (2014). Safety and efficacy of addition of VEGFR and EGFR-family oral small-molecule tyrosine kinase inhibitors to cytotoxic chemotherapy in solid cancers: A systematic review and meta-analysis of randomized controlled trials. Cancer treatment reviews.

[88] Ebos JM, Lee CR, Cruz-Munoz W, Bjarnason GA, Christensen JG, Kerbel RS (2009). Accelerated metastasis after short-term treatment with a potent inhibitor of tumor angiogenesis. Cancer cell.

[89] Paez-Ribes M, Allen E, Hudock J, Takeda T, Okuyama H, Vinals F (2009). Antiangiogenic therapy elicits malignant progression of tumors to increased local invasion and distant metastasis. Cancer cell.

[90] Sennino B, Ishiguro-Oonuma T, Wei Y, Naylor RM, Williamson CW, Bhagwandin V (2012). Suppression of tumor invasion and metastasis by concurrent inhibition of c-Met and VEGF signaling in pancreatic neuroendocrine tumors. Cancer discovery.

